# Bovine Serum Albumin Rejection by an Open Ultrafiltration Membrane: Characterization and Modeling

**DOI:** 10.3390/membranes14010026

**Published:** 2024-01-21

**Authors:** Eric Suryawirawan, Anja E. M. Janssen, Remko M. Boom, Albert van der Padt

**Affiliations:** 1 Food Process Engineering Group, Wageningen University, P.O. Box 17, 6700 AA Wageningen, The Netherlands; remko.boom@wur.nl (R.M.B.); albert.vanderpadt@wur.nl (A.v.d.P.); 2 FrieslandCampina, P.O. Box 1551, 3800 BN Amersfoort, The Netherlands

**Keywords:** protein transport, ultrafiltration, solution–friction model, general rejection equation

## Abstract

The classic application of ultrafiltration (UF) is for the complete retention of proteins, and in that situation, the transport behavior is well established. More open membranes with fractional retention are used when separating different proteins. However, protein transport has not been well documented yet in the literature. The bovine serum albumin (∼69 kDa) observed rejection ranges from 0.65 to 1 using a 300 kDa molecular weight cut-off membrane at different pH, ionic strength, and pressure. We demonstrated that, especially with open UF, the transport of proteins through the membrane is dominated by advection, with insignificant diffusion effects (*p* value > 0.05). We showed that with open UF, retention is not only caused by size exclusion but also to a large extent by electrostatic interactions and oligomerization of the proteins. Mass transfer in the polarization layer was relatively independent of the pH and ionic strength. It was underestimated by common Sherwood relations due to a relatively large contribution of the reduction in the flow turbulence near the membrane by the removal of fluid through the membrane. We propose a model that allows relatively quick characterization of the rejection of proteins without prior knowledge of the pore sizes and charges based on just a limited set of experiments. Therefore, protein rejection with the open UF system can be targeted by tuning the processing conditions, which might be useful for designing protein fractionation processes.

## 1. Introduction

Ultrafiltration (UF) has been successfully applied for the separation and purification of biomolecules. For classic use cases, such as concentration, buffer exchange, and desalination, UF membranes with (almost) full retention of the macromolecules are used. Rigorous models are available to describe the volumetric flux, and the retention of macromolecules is generally assumed to be 100% in these applications [1,2,3]. However, there is increasing interest in separating proteins with UF. Many proteins are relatively similar in size, and therefore their retention is also expected to be similar. To achieve separation, moderate retention is required by using a UF membrane that is permeable to the solutes (open UF), and the use of separation mechanisms other than size exclusion could add to the selectivity of the process [4,5,6].

Even though many studies discuss improving protein fractionation with UF by modifying the membrane or the system configuration [5,7,8], the framework for quantitatively describing the transport of a protein through open UF membranes has not yet been well described due to the complexity attributed to the simultaneous action of multiple driving forces and the presence of several components in the system [9]. Proteins may have a different charge at different values of pH and ionic strength, which complicates a full description. Moreover, proteins may form oligomers under certain conditions such as high protein concentration and proximity to the protein’s pI [10,11]. Therefore, a description of protein transport with a model that allows this complexity to be lumped into a parameter may be useful.

The transport of a solute in reverse osmosis (RO), nanofiltration (NF) and electrodialysis (ED) has been described by Biesheuvel et al. [12] by using a solution–friction model that includes advection, diffusion, and electrostatic interaction. Although NF and UF are different in many ways, the approach may be adapted to describe the transport of charged particles in open UF. Due to the high flux through an open UF membrane [13], we hypothesize that diffusion through the membrane pores is not significant and that electrostatic interactions are important.

We describe protein permeation through an open UF membrane, evaluate the importance of diffusion and advection, and assess common Sherwood relations for the mass transfer rates in the polarization layer. We used bovine serum albumin (BSA; molecular weight [MW], ∼69 kDa) as the model protein, an open UF membrane (molecular weight cutoff [MWCO], 300 kDa) as the model membrane, and conducted experiments at pH 4.9 and 7 and various ionic strengths.

## 2. Theoretical Background

**Figure 1:**
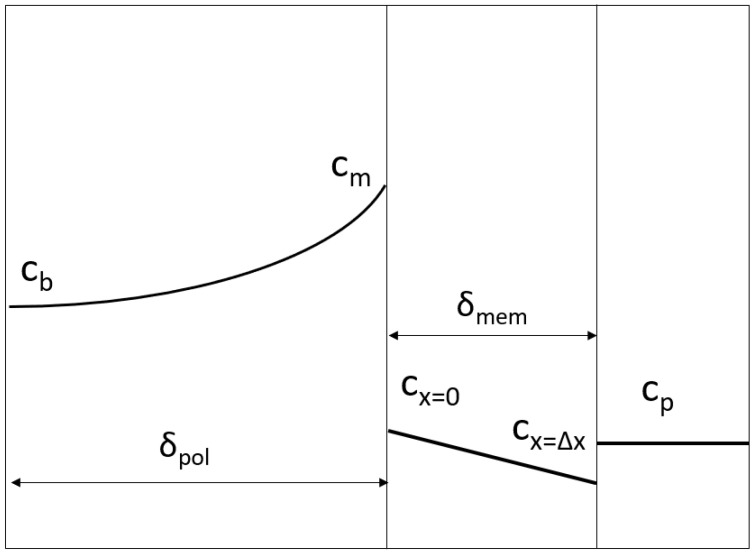
The concentration profile of a solute during ultrafiltration in the retentate, membrane and permeate, with the thickness of the polarization layer $\delta_{pol}$ and the length of the active layer of membrane $\delta_{mem}$ . $c_{b}$ is the solute concentration at retentate bulk, $c_{m}$ is the solute concentration at the membrane surface, $c_{x=0}$ is the concentration at the membrane entrance, $c_{x=\Delta x}$ is the concentration at the membrane exit, and $c_{p}$ is the concentration at the permeate stream.

The mass transfer of a solute through a UF membrane involves sequential processes: (1) mass transfer through the concentration polarization layer, (2) solute partitioning into the pores, (3) permeation through the selective (top) layer of the membrane, and (4) release into the permeate on the other side (Figure 1). Solutes that are retained by the membrane accumulate on the surface of the membrane, leading to polarization (process 1). The resulting concentration gradient at the membrane surface causes back-diffusion of the solutes towards the retentate bulk. The thickness of this polarization layer depends on the hydrodynamics in the feed channel and the properties of the solution.

Processes 2–4 involve various strategies to predict partitioning and protein transport through membranes. Due to the complexity of proteins as a solute, an ab initio model will require many parameters, which cannot be assessed independently, and therefore would leave too many parameters to fit in the experimental results. For practical purposes, a prediction of protein transport through UF can be obtained using a limited set of experimental data using a model that combines some of the complexity into a few lumped parameters. We here derive such a model that allows us to find this balance between experimental characterization and model description.

### 2.1. Generalized Solute Retention Equation

The transport of neutral solutes through the selective layer of the membrane can be described by Equation (1) [12]. Even though protein is a charged solute, a protein is here regarded as a large neutral solute because protein moves together with its counterions; at sufficient ionic strength and with a membrane that does not retain any small ions such as in UF, this results in regular diffusion [14]. Under these conditions, we can assume that the electrostatic interaction is incorporated in the relevant parameters (mass transfer and sieving coefficients). In this case, the transport of solute *i* is assumed to be independent of the transport of other solutes:

(1) $$J_{i}=K_{f,i}c_{mem,i}v_{w}-K_{f,i}D_{m,i}\frac{dc_{mem,i}}{dx}$$

$$J_{i}=J_{v}c_{p,i}$$

where $J_{i}$ [mol m^−2^ s^−1^] is the overall solute flux, which is a combination of solute transport due to advection (first term) and diffusion (second term). It can be calculated experimentally using the volumetric flux ( $J_{v}$ [m s^−1^]) and the concentration in the permeate $C_{p,i}$ [mol m^−3^]. The velocity of water $v_{w}$ [m s^−1^] is approximately the same as the volumetric flux $J_{v}$ due to the low solute concentration in the permeate. They are used interchangeably throughout the discussion; $v_{w}$ is used for only calculated values and $J_{v}$ when the measurements were carried out. The calculation of the advection part incorporates the interaction of the solute with the pore. This requires a friction factor between the solute and the membrane/pore wall ( $K_{f,i})$ [−], and requires the concentration inside the membrane pores ( $c_{mem,i}$ [mol m^−3^]). This concentration differs from the concentration outside the pores due to exclusion and affinity effects. For the transport due to diffusion, the calculation requires the diffusion coefficient of the solute in the pores ( $D_{m,i}$ [m^2^ s^−1^]), again the friction factor mentioned earlier, and the concentration gradient along the length of the pores ( $dc_{mem,i}/dx$ [mol m^−4^]). A friction factor of zero implies that there can be no transport of solute through the membrane, and a friction factor of 1 means there is no friction between the solute and the membrane. A similar equation can be used for the polarization layer by setting $K_{f,i}=1$ and applying the appropriate diffusion coefficient for the liquid phase and concentration at the retentate side. In an extended theory where the pore space is partially occupied by the solutes, $K_{f,i}$ can be replaced with hindrance factors for advection/convection ( $K_{c,i}$ ) and diffusion ( $K_{d,i}$ ) (Equation (2)) [12]:

(2) $$J_{i}=K_{c,i}c_{mem,i}v_{w}-K_{d,i}D_{m,i}\frac{dc_{mem,i}}{dx}$$

Equation (2) can be integrated across the length of the pores through the active layer of the membrane ( $\delta_{mem}$ [m]), giving:

(3) $$Pe_{m,i}=\frac{K_{c,i}\delta_{mem}v_{w}}{K_{d,i}D_{m,i}}=\frac{v_{w}}{k_{m,i}}=-\ln\left(\frac{J_{i}-v_{w}K_{c,i}c_{x=0}}{J_{i}-v_{w}K_{c,i}c_{x=\Delta x}}\right)$$

$$k_{m,i}=\frac{K_{d,i}D_{m,i}}{K_{c,i}\delta_{mem}}$$

The quantity $Pe_{m,i}$ is the Peclet number for component *i* in the membrane. Unlike the mass transfer coefficient definition in the polarization layer, the mass transfer coefficient of the solute in the membrane $k_{m,i}$ is also determined by the hindrance factors ( $K_{c,i}$ and $K_{d,i}$ ) in addition to the diffusion coefficient of solute *i* in the membrane ( $D_{m,i}$ ) and the thickness of the membrane ( $\delta_{mem}$ ). The concentrations $c_{x=0}$ and $c_{x=\Delta x}$ are the internal concentrations at the entrance and exit sides of the pores, respectively. We can relate these internal concentrations to the outside concentrations with a partition coefficient $\Phi_{i}=c_{x=0,i}/c_{m,i}$ , which incorporates the effects of affinity and exclusion due to size, charge, or other interactions.

In the polarization layer, Equation (1) can be integrated across the thickness of the polarization layer ( $\delta_{pol}$ ) with $K_{f,i}=1$ :

(4) $$Pe_{d,i}=\frac{v_{w}\delta_{pol}}{D_{i}}=\frac{v_{w}}{k_{dbl,i}}=-\ln\left(\frac{J_{i}-v_{w}c_{b,i}}{J_{i}-v_{w}c_{m,i}}\right)$$

$$k_{dbl,i}=\frac{D_{m,i}}{\delta_{pol}}$$

where $Pe_{d,i}$ is the Peclet number of component *i* in the concentration polarization layer, which can also be calculated with the mass transfer coefficient in the polarization layer $Pe_{d,i}=v_{w}/k_{dbl,i}$ . $c_{b,i}$ is the concentration in the retentate bulk, and $c_{m,i}$ is the solute concentration on the membrane surface. After simplification and rearrangement, the expression for the solute flux for a neutral solute in the membrane can be written as:

(5) $$J_{i}=\frac{K_{c,i}\Phi_{i}v_{w}\left(c_{p,i}\exp\left(-Pe_{m,i}\right)-c_{m,i}\right)}{\exp(-Pe_{m,i})-1}$$

and the expression for solute flux in the polarization layer is:

(6) $$J_{i}=\frac{v_{w}\left(c_{m,i}\exp\left(-Pe_{d,i}\right)-c_{b,i}\right)}{\exp(-Pe_{d,i})-1}$$

The sieving coefficient $\sigma_{i}$ is related to the partition coefficient and the friction factor by:

(7) $$\sigma_{i}=1-K_{c,i}\Phi_{i}$$

Following Biesheuvel et al. [12] and Starov and Churaev [15], by combining these equations, a generalized solute rejection equation can be obtained that includes the effect of concentration polarization (Equation (8); see Appendix A.1 for the derivation):

(8) $$R_{obs,i}=1-\frac{1-\sigma_{i}}{1-\sigma_{i}+\exp\left(-Pe_{d,i}\right)\left(1-\exp\left(-Pe_{m,i}\right)\right)\sigma_{i}}$$

For mass transfer in UF, Rohani and Zydney [13] assumed that due to the high Peclet number (high flux) in these systems, protein transport is dominated by advection. If we accept this assumption ( $K_{d,i}D_{m,i}\ll K_{c,i}v_{w}\Delta x_{mem}$ ), then the term $\exp(-Pe_{m,i})\to 0$ and the rejection equation without diffusion through the membrane can be obtained (Equation (9); Appendix A.2):

(9) $$R_{obs,i}=1-\frac{1-\sigma_{i}}{1-\sigma_{i}+\exp\left(-Pe_{d,i}\right)\sigma_{i}}$$

The parameters $\sigma_{i}$ , $Pe_{d,i}$ , and $Pe_{m,i}$ in Equations (8) and (9) can be adapted to match the experimental results.

### 2.2. Mass Transfer Coefficient in the Polarization Layer

The mass transfer coefficients in the polarization layer ( $k_{dbl,i}$ ) [m s^−1^] in crossflow UF have been studied by van den Berg et al. [16]. The traditional approach for estimating $k_{dbl,i}$ is to use a Sherwood equation (Equation (10)):

(10) $$Sh=\frac{d_{h}}{\delta }=\frac{k_{dbl,i}d_{h}}{D}=ARe^{m}Sc^{n}$$

where $d_{h}$ [m] is the characteristic diameter of the system, $k_{dbl,i}$ is the mass transfer coefficient, *D* is the diffusion coefficient, Re [-] is the Reynolds number, Sc [-] is the Schmidt number, and *A*, *m*, and *n* are dimensionless fitted parameters. The characteristic diameter, Reynolds number, and Schmidt number were calculated as follows:

(11) $$Re=\frac{\rho v_{r}d_{h}}{\eta }$$

(12) $$Sc=\frac{\eta }{\rho D}$$

(13) $$d_{h}=\frac{4\varepsilon }{\frac{2}{h}+\left(1-\varepsilon \right)S_{v,p}}$$

For the Re and Sc calculations, ρ [kg m^−3^] is the density of the solution, $v_{r}$ [m s^−1^] is the crossflow velocity in the retentate channel, and η [Pa s] is the viscosity of the solution. For the hydraulic diameter $d_{h}$ , ε [-] is the porosity of the spacer, *h* [m] is the height of the channel, and $S_{v,p}$ [m^−1^] is the specific surface of the spacer.

## 3. Materials and Methods

### 3.1. Materials

Lyophilized BSA with ≥96% purity was used. NaCl (≥99.5% purity) was used to adjust the ionic strength of the solution. Solutions of 2 M NaOH or HCl (≥99% purity) were used to adjust the pH of the solution. All chemicals were purchased from Sigma-Aldrich (Steinheim, Germany). Milli-Q water (ultrapure water) obtained from Millipak 40 Express Filter with a pore size of 0.22 μ m was used to prepare all the solutions in this study.

### 3.2. Membrane and Setup

A polyethersulfone UF membrane (Synder Filtration, Vacaville, CA, USA) in a spiral wound configuration was used in all experiments (Table 1). The experiments were performed with a pilot-scale membrane unit that allowed the control and registration of the flow, pressure, and temperature of both permeate and retentate streams. The system temperature was manually controlled with an external water bath connected to the retentate’s recirculation loop. The setup configuration is described in more detail by Aguirre Montesdeoca et al. [17].

### 3.3. Experiment

BSA solutions (0.5% (*w*/*v*)) were prepared at different pH and ionic strengths (pH 7: 0, 0.08, 0.15, and 0.2 M NaCl; pH 4.9: 0 and 0.2 M NaCl). After dissolving the solutes, the pH of the solution was measured with a SevenMulti pH meter and then adjusted to the desired pH by adding aliquots of 2 M NaOH or HCl solutions. The prepared solutions were then filtered with a Whatman glass microfiber GF/D (GE Healthcare Life Sciences, Amersham, UK) to remove any aggregates or undissolved materials. The solutions were used immediately or stored overnight at 4 °C. Although the solution with 0 M NaCl was prepared without added salt, there is a very small amount of ions present in the prepared solution (≤2 mM), but their presence can be considered insignificant.

Before protein filtration, the setup was conditioned by circulating a salt solution with the same pH and ionic strength as the protein solution for at least 10 min. The filtration was performed at 25 °C with a constant cross-flow velocity of 0.167 m/s. The filtration was performed at 0.2, 0.5, 0.8, 1.5, and 3 bar transmembrane pressure. The solution was circulated for 45 min for each pressure, assuming a quasi-steady state condition had been reached after this time. Samples from the retentate and permeate streams were collected to measure the solute concentrations in duplicate.

### 3.4. Analytical Methods

High-performance liquid chromatography (HPLC) was used to measure the BSA concentrations in the retentate and permeate streams. Specifications of the columns and processing conditions are shown in Table 2.

### 3.5. Fitting Equations

The experimental observed rejection of BSA can be calculated from the measured BSA concentration at the retentate and permeate for each set of conditions. The parameters then can be fitted to the experimental values with Equation (8) for the general rejection equation and with Equation (9) for the rejection equation without diffusion through the membrane by minimizing the sum of the squared residuals of the observed rejections (Equation (14)). Fitting was performed by using the fitnlm function in MATLAB2018b (MathWorks), available in the statistics and machine learning toolbox:

(14) $$\min f\left(x\right)={\left(R_{obs,exp}-R_{obs,calc}\right)}^{2}$$

The fit of the equations can be compared using F statistics, which can be calculated by:

(15) $$F=\frac{\left(SS_{1}-SS_{2}\right)/\left(df_{1}-df_{2}\right)}{SS_{2}/df_{2}}$$

$SS_{1}$ and $df_{1}$ are the sum of square residuals and the degree of freedom of an equation with fewer parameters that would have higher values than the equation with more parameters ( $SS_{2}$ and $df_{2}$ ). The *p* values were calculated using the 1-fcdf function in MATLAB2018b with $\left(df_{1}-df_{2}\right)$ and $df_{2}$ as the numerator and denominator, respectively.

## 4. Results and Discussion

### 4.1. Comparison of the Generalized Solute Rejection Equation with and without Diffusion

**Figure 2:**
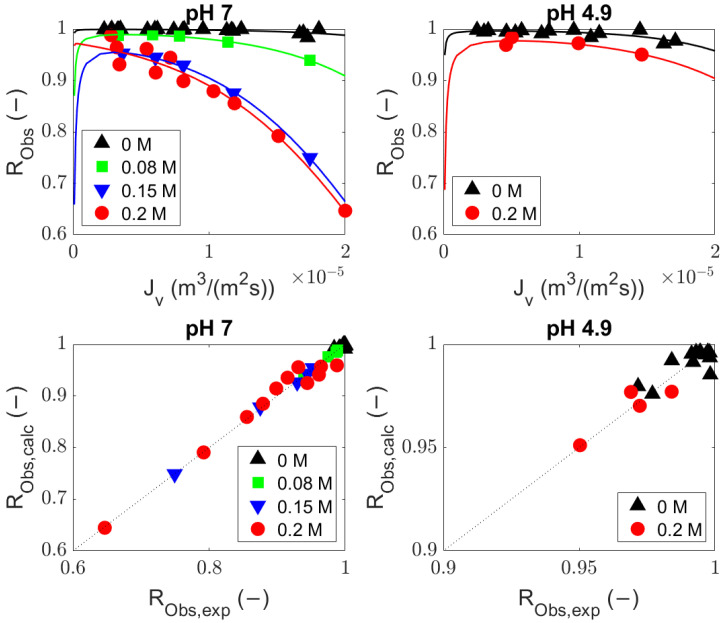
Fitted bovine serum albumin observed rejection at pH 7 and 4.9 with the general rejection equation (Equation (8)); average adj-*r*^2^ = 0.76.

**Figure 3:**
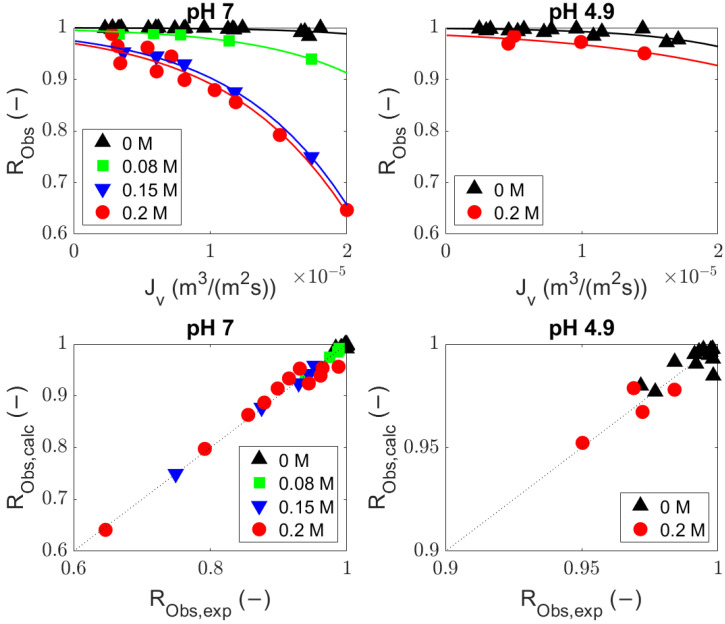
Fitted bovine serum albumin observed rejection at pH 7 and 4.9 with the rejection equation without diffusion (Equation (9)); average adj-*r*^2^ = 0.75.

Experiments were conducted using BSA (MW, ∼69 kDa) solutions with a UF membrane (MWCO, 300 kDa) at different values of pH and ionic strength (pH 7: 0, 0.08, 0.15, and 0.2 M NaCl; pH 4.9: 0 and 0.2 M NaCl). The rejection equations including (Equation (8)) and excluding (Equation (9)) diffusion were compared with the observed rejections from the experiments (Figure 2 and Figure 3, volumetric flux and observed rejection of BSA values are available in Appendix B.1) by fitting the sieving coefficient ( $\sigma_{i}$ ) and the effective mass transfer coefficients of the solute in the polarization layer ( $k_{dbl,i}$ ) and in the membrane ( $k_{m,i}$ ) (values are given in Appendix B.2). The predicted values from both equations (Figure 2 and Figure 3) are comparable and describe the experimental data well.

**Figure 4:**
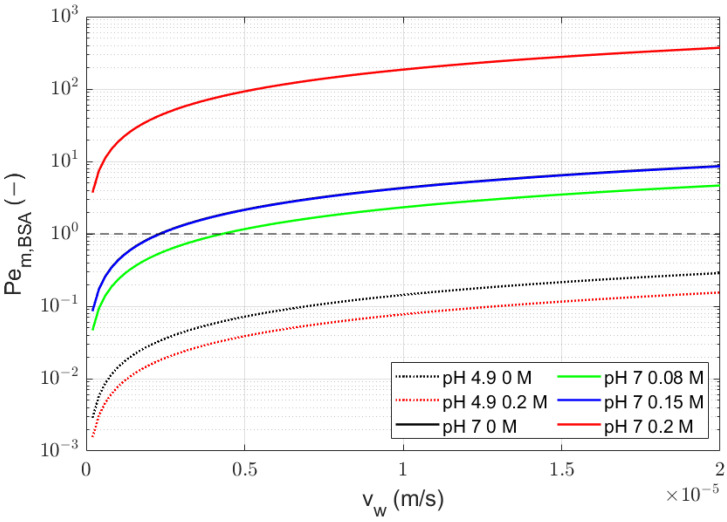
$Pe_{m,i}$ profile with arbitrary flux with fitted $k_{m,i}$ values. The calculated value of $Pe_{m,i}$ for pH 7 and 0 M is overlaid by the line for pH 7 0.15 M. The black dashed line (– –) represents $Pe_{m,i}=1$ , above which advection is dominant over diffusion.

The $k_{m,BSA}$ values found with Equation (8) were used to assess the value of $Pe_{m,BSA}$ as a function of the volumetric flux $v_{w}$ (Figure 4). Its value at pH 7 and high ionic strength (0.2 M NaCl) is 10 or higher, which indicates that the contribution of the diffusion to the transport through the membrane is negligible. For a lower ionic strength at pH 7, $Pe_{m,BSA}$ is smaller but still >1. Here, the contribution from diffusion becomes progressively less significant at higher volumetric flow rates. At pH 4.9, the situation is somewhat different because then, $Pe_{m,BSA}$ is <1. These lower $Pe_{m,BSA}$ values indicate a higher diffusion coefficient of BSA in the membrane near its pI compared with pH 7. This lower diffusion coefficient of BSA in the membrane at pH 7 might be caused by electrostatic interactions between BSA and the membrane pore wall that hinder the movement of the solute. This is in agreement with Dechadilok and Deen [18], who concluded that charged particles have a slower diffusivity in a membrane pore compared with a neutral solute of the same size. However, the $r^{2}$ values for both equations for BSA at pH 4.9 were low (Table 3). The reliability of the parameters with both equations is therefore low and should be considered with this in mind.

**Figure 5:**
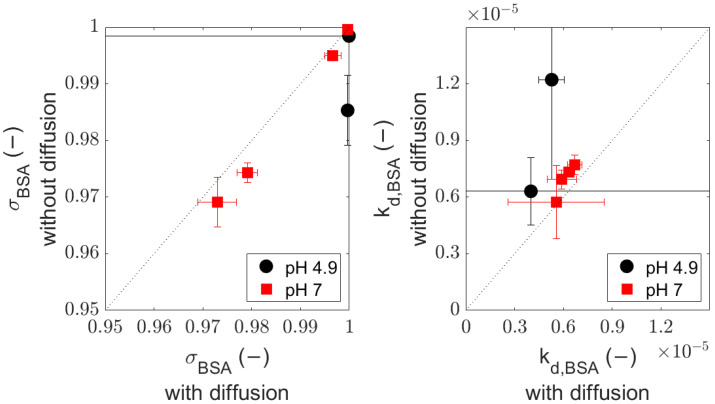
Fitted $\sigma_{BSA}$ (**left**) and $k_{dbl,BSA}$ (**right**): comparison with and without diffusion through the membrane.

The $\sigma_{BSA}$ and $k_{dbl,BSA}$ values at pH 7 from both models are comparable and have similar values, which indicates that the diffusion inside the pores does not contribute significantly to membrane permeation. However, with pH 4.9, omission of the diffusion in the pores results in different $\sigma_{BSA}$ and $k_{dbl,BSA}$ values. This is illustrated in Figure 5.

One can see that the reliability for the parameters is poor, and F tests comparing the two models statistically (Table 3) shows that there is no significant difference between these equations. Therefore, although we cannot conclude that diffusion is unimportant at this pH, we can say that its inclusion is not required for describing the rejections.

**Figure 6:**
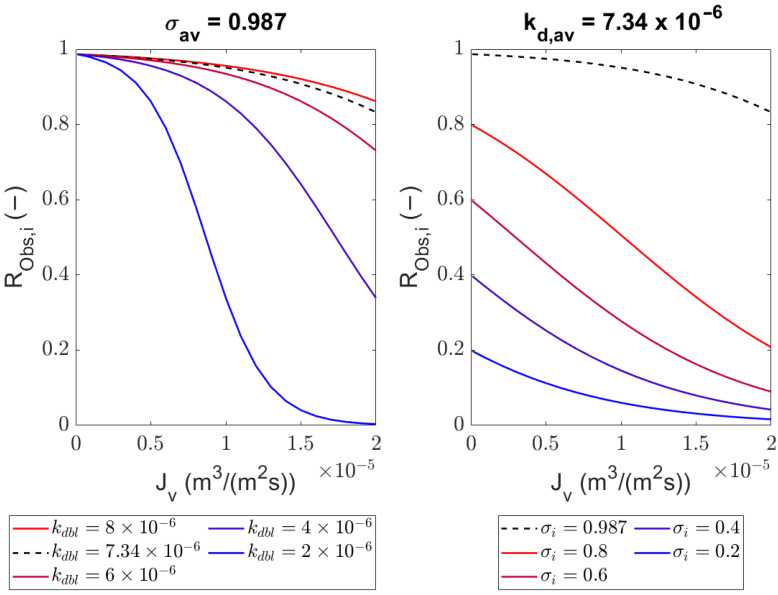
$R_{obs,i}$ profile with arbitrary $k_{dbl,i}$ (**left**) and $\sigma_{i}$ (**right**) as a function of $v_{w}$ with the averaged value of $k_{dbl,BSA}$ and $\sigma_{BSA}$ from the advection-dominated rejection equation fitting (Equation (9)).

If we accept the model without the contribution of diffusion to permeation, we can predict the rejections as a function of the volumetric flow rate or the transmembrane pressure as long as we avoid cake layer formation (leading to limiting flux behavior) (Figure 6). The sieving coefficient $\sigma_{i}$ represents $R_{obs,i}$ at $v_{w}\to 0$ (thermodynamic equilibrium), which is also its maximum value. The value of the mass transfer coefficient $k_{dbl,i}$ represents the kinetics of solute *i* in the polarization layer. A larger $k_{dbl,i}$ implies a thinner polarization layer, which can be achieved by a larger crossflow rate or a more efficient design of the spacer. Figure 6 shows that we can achieve both high and intermediate retentions by choosing the right conditions ( $v_{w}$ by the transmembrane pressure and $k_{dbl,i}$ by the crossflow conditions).

The separation of proteins requires intermediate retentions to obtain selectivity of one protein over another, and therefore our model shows that the membrane used in this work is suitable for this aim, even though its nominal MWCO is 300 kDa. This indicates that the separation mechanism is not only size exclusion but is also probably related to charge effects because MWCO values are generally determined with uncharged solutes.

Observations from the rejections of BSA also demonstrate the effect of electrostatic interaction during the UF of protein. With a preliminary estimate using the solute MW and the membrane MWCO, a prospective membrane user would consider the steric exclusion (partitioning only based on the solute to pore size ratio), which can be estimated with the Ferry equation ( $K_{i}={\left(1-r_{s}/r_{p}\right)}^{2}$ ) [19,20]. With an approximate membrane pore size of 5.69 nm (assuming the pore size is the same as the size of folded globular protein with a MW of 300 kDa [21]) and the theoretical size of BSA (3.48 nm), the ratio of the solute that can enter the membrane pore is about 15%, which roughly translates to 85% rejection when no concentration polarization effect is present. However, we observed high rejections (>90%) at pH 7 with lower ionic strength, and the rejection becomes closer to the approximate rejection value in the high ionic strength condition. This behavior indicates that the electrostatic interaction plays a role in determining protein rejection, and the rejection of a large charged solute such as protein is higher than that of a neutral solute with the same size due to the electrostatic interactions.

### 4.2. Sieving Coefficients

**Figure 7:**
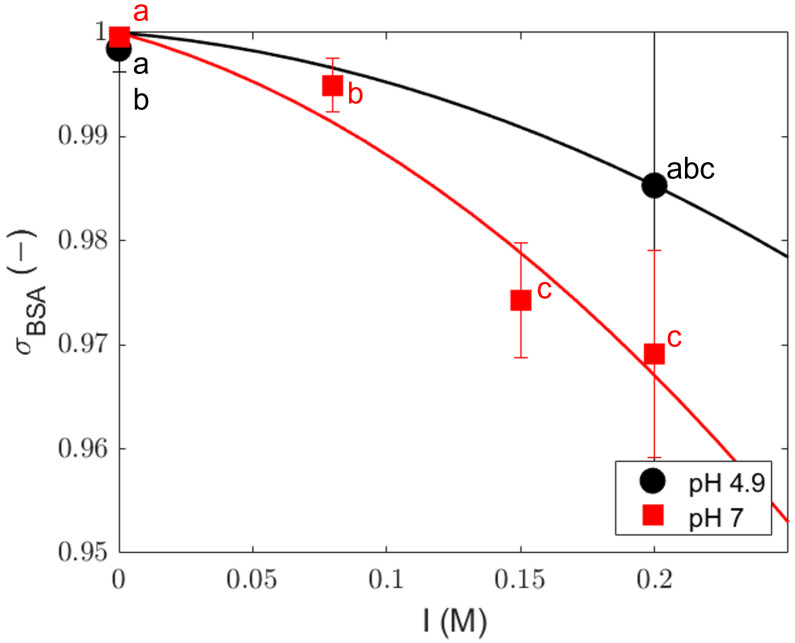
Fitted $\sigma_{BSA}$ with the advection-dominated rejection equation; error bars represent a 95% confidence interval. A marker followed by different lower-case letters indicates a significant difference within a 95% confidence interval. The lines are only a visual guide.

Our conclusion that charge effects may dominate the retention behavior in our system justifies a closer assessment. At low ionic strengths, the electrostatic repulsion between the proteins and the membrane pore walls creates an exclusion effect, which leads to a high sieving coefficient $\sigma_{i}$ . At higher ionic strengths, these electrostatic interactions are screened, and the sieving coefficient decreases (Figure 7); therefore, more solute passes through the membrane. This sieving coefficient $\sigma_{i}=1-\Phi_{i}K_{f,i}$ (Equation (7)) is different from the observed sieving ( $S_{obs}=c_{per}/c_{ret}$ ), which is used to describe the solute ratio in the permeate and retentate instead of the observed rejection ( $R_{obs}=1-c_{per}/c_{ret}$ ). Given the discrepancy between the nominal membrane MWCO of 300 kDa and the MW of BSA (69 kDa), we would expect low retention for the protein. Regardless, the system showed high intrinsic rejection ( $R_{Obs}>0.9$ ) at pH 4.9 ( at both 0 M and 0.2 M NaCl) and pH 7 (at 0 M NaCl). The high rejections found at pH 4.9 and 7, and ionic strengths from 0 to 0.2 M, translate into sieving coefficients $\sigma_{i}$ that are close to 1 and decreasing at higher ionic strengths. The lower values at a higher ionic strength indicate that the retention is caused by electrostatic effects. The low diffusion coefficient at pH 7 and the lower value at pH 4.9 are in accordance with this; the protein charge at pH 7 causes stronger friction with the pore walls than at a pI of 4.9.

In our system, the rejection of the protein therefore occurs due to membrane–protein electrostatic interactions. Although the influence of size exclusion may not be dominant, ionic interactions are probably quite important. We see in Figure 7 that at higher ionic strengths, the sieving coefficient decreases as expected for electrostatic exclusion. The effect is stronger at pH 7 than at a pI of 4.9 because the protein has a stronger net charge at pH 7.

The electrolyte–membrane interaction could be described by the Donnan potential at the membrane interface as discussed by Bowen and Welfoot [22] for the transport of ions, or by using a partitioning relation based on the Poisson–Boltzmann relation as described by Smith and Deen [23]. Both of these require more knowledge of the membrane properties, such as membrane charge or pore size, which are generally not available in practice. The solute partition coefficient $\Phi_{i}$ can be defined as [23]:

$$\Phi_{i}=\frac{c_{x=0,i}}{c_{m,i}}={\left(1-\frac{r_{s,i}}{r_{p}}\right)}^{2}\exp\left[\frac{-E_{T}}{k_{B}T}\right]$$

where $r_{s,i}$ is the solute radius, $r_{p}$ is the pore radius, $E_{T}$ is the total interaction energy between solute and membrane, $k_{B}$ is the Boltzmann constant, and *T* is the absolute temperature. Based on this theory, one can expect a lower $\Phi_{i}$ (high $\sigma_{i}$ ) with a solute with a larger charge number and at a lower ionic strength condition due to the larger electrostatic interaction, which contributes to the interaction between the solute and membrane.

The high rejections of BSA at pH 4.9 at any ionic strength and pH 7 at 0 M NaCl may be partly caused by the self-oligomerization of BSA under these conditions, which increases the size of the solute and influences the $\sigma_{i}$ . Bhattacharya et al. [11] identified various oligomeric states of serum albumins at concentrations as low as 10–150 μ m at pH 7.4. Pohl et al. [10] showed that the oligomerization of human interferon alpha-2a depends on the pH, protein concentration, and ionic strength of the solution. They observed more oligomerization when approaching the protein’s pI and at higher protein concentrations, and the addition of salt reduced the aggregation. An increase in ionic strength thus increases the permeation of the solute both by reducing the solute–membrane electrostatic interaction and by preventing or reducing oligomerization. However, at a very high salt concentration, the protein can again aggregate [24].

The effect of pH on the rejection of BSA is not straightforward. Proteins have a higher charge when the pH of the solution is further from the pI of the protein, and the tendency of the protein to form oligomers decreases at higher charge numbers. When the charge signs of the membrane and protein are the same, there is electrostatic repulsion between the solute and the membrane, which contributes to the protein rejection. When the charges of the protein and membrane are opposite, the electrostatic interactions between the membrane and the protein are attractive and may even induce extensive fouling of the membrane. The protein will have the strongest tendency to precipitate at its pI, which may then foul and block the membrane, effectively making UF impractical. In a protein–surface adsorption study by Rabe et al. [24], attractive electrostatic forces increased the adsorption of protein to the surface, with maximal adsorption at the pI of the protein. Therefore, an electrostatic attractive force may increase the permeation of protein, but it also increases protein adsorption at the membrane surface, which can result in a change of the rejection mechanism and a strong reduction in flux.

### 4.3. Mass Transfer Coefficient in the Polarization Layer

**Figure 8:**
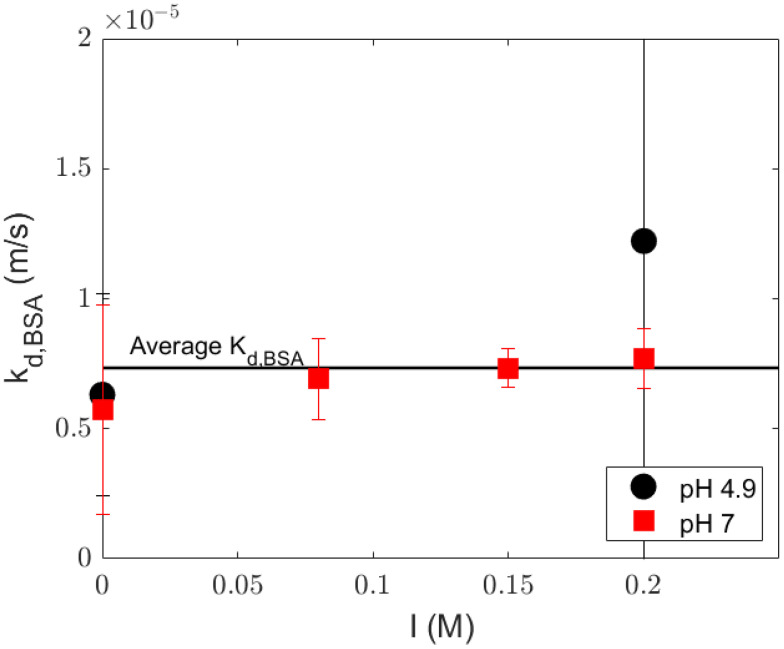
Fitted $k_{dbl,BSA}$ with the advection-dominated rejection equation; error bars represent 95% confidence interval. Lines are only a guide for the eye.

The diffusion rates of the charged components in the polarization layer are dependent to some degree on the pH and ionic strength, but this kinetic effect is minor compared to the effects of the thermodynamic exclusion by size and charge in the membrane pores. The mass transfer coefficient of a solute is mostly dependent on the hydrodynamics on the retentate stream and the properties of the solute. Because the experiments were performed with the same solutes, membrane, and membrane module, and the crossflow velocity was not changed, we expect that the mass transfer coefficient is relatively constant for different values of pH and ionic strength. This was confirmed by the experimentally determined mass transfer coefficients (Figure 8).

Because there is no significant difference in the mass transfer coefficient at different values of pH and ionic strength, the average mass transfer coefficient in the polarization layer ( $7.34\times 10^{-6}$ m/s) can be used. Alternatively, the mass transfer coefficient can be estimated via Sherwood relations, which would leave only the sieving coefficient as an unknown parameter in the equation. Several relations from the literature were compared with the average values of the fitted mass transfer coefficient. Most of these relations underestimated the mass transfer coefficient (Table 4). Even though the relation from Schock and Miquel [25] was reported to be inadequate due to inaccuracies [26], it gave the closest value to the average mass transfer coefficient we found from the experiments. Van den Berg et al. [16] suggested the Graetz–Leveque relation for laminar flow conditions and the Harriott–Hamilton relation for solutions with turbulent flow and a high Schmidt number, but these relations did not give a correct estimation. The most recent studies on Sherwood relations were performed by Bandini and Morelli [26] and Shi et al. [27] for a small lab-/pilot-scale 1812 spiral wound module. Those relations also resulted in inadequate estimations for our system, which might be because these authors used an NF spiral wound system. The mass transfer relations derived from NF or RO can be inaccurate due to large differences in the solute Schmidt numbers: for RO or NF of salt solutions, the Sc < 1000, whereas in the UF of protein solutions, Sc > 10,000 [28].

The so-called suction effect might also explain this higher experimental mass transfer coefficient compared with those obtained from the literature. In a fluid mechanics study by Belfort and Nagata [29], the onset of turbulence in the retentate stream was shifted from a Reynold number of 2100 to about 4000 in a porous tube compared with a non-porous tube because the removal of solvent from the membrane surface through the membrane also attenuates the eddies near the surface. Therefore, the laminar flow pattern near the membrane interface is stabilized, and the mass transfer coefficient is enhanced. Because our study was performed with an open membrane that allows relatively large fluxes, we expect that the suction effect is significant in our experiments. This results in higher mass transfer coefficients compared with estimations based on the mass transfer coefficients from the literature (Table 4). The thickness of the boundary layer is smaller due to the higher mass transfer coefficient, which leads to less accumulation of solutes on the membrane surface. Therefore, the calculated concentrations of solutes at the membrane interface would be overestimated by using Sherwood relation in this system, leading to inaccuracies in the rejection prediction of a solute, especially in a system with relatively high fluxes. In addition, it is difficult to quantify the suction effect with the value from the Sherwood number relation because they are not derived with the same module geometry and we did not vary $k_{dbl,i}$ with the volumetric flux in our models.

**Table 4:** Sherwood relation comparison with the average fitted mass transfer coefficient.

Sherwood Relation	$k_{\mathrm{dbl}}$ (m/s)	Adj-*r*^2^	Source
Average fitted mass transfer	7.34 $\times \;10^{-6}$	0.74	Spiral wound 1812 UF Protein
(this work)			
Schock and Miquel [25]	4.27 $\times \;10^{-6}$	0.50	Spiral wound 2540 RO and UF salt
$Sh=0.065Re^{0.875}Sc^{0.25}$			
Graetz–Leveque equation [16]	2.32 $\times \;10^{-6}$	0.02	Heat–mass transfer analogy [30]
$Sh=1.86Re^{0.33}Sc^{0.33}{\left(d_{H}/L\right)}^{0.33}$			
Harriot–Hamilton equation [16]	1.97 $\times \;10^{-6}$	−2.65	Turbulent flow in pipe [31]
$Sh=0.0096Re^{0.91}Sc^{0.35}$			
Bandini and Morelli [26]	3.15 $\times \;10^{-4}$	0.03	Spiral wound 1812 NF dextrose
$Sh=0.016Re^{0.8}Sc^{1/3}$			
Shi et al. [27]	2.67 $\times \;10^{-6}$	0.12	Spiral wound 1812 organic solvent NF
$Sh=0.075Re^{0.61}Sc^{0.33}$			

**Figure 9:**
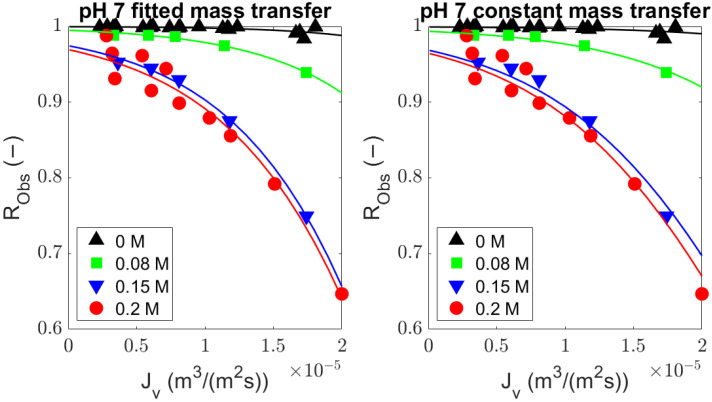
Comparison between a model where the mass transfer coefficients are fitted for each individual condition (**left**) and where the mass transfer coefficient for each component is taken to be the same for all conditions (**right**).

The model for the advection–dominated rejection equation (Equation (9)) only requires two fitting parameters (both $k_{dbl}$ and $\sigma_{i}$ ) or just one with only $\sigma_{i}$ fitted, with $k_{dbl}$ values obtained from Table 4; the parameter values from both strategies are given in Appendix B, Table A2. F statistics, as shown in Table 5, show that the inclusion of the mass transfer coefficient of the polarization layer as a fitting parameter improves the quality of the prediction significantly. We can reduce the number of parameters by assuming that the mass transfer coefficient does not depend on the ionic strength or the pH. We can then find the $\sigma_{i}$ value for each condition. Table 5 shows that the mass transfer coefficient value of $7.34\times 10^{-6}$ m/s resulted in good descriptions in the observed rejection profiles. The result is comparable with the results with the one-parameter model (Figure 9). The value of $\sigma_{i}$ fitted for each Sherwood relations and their adj- $r^{2}$ is reported in Table 6. Therefore, the pH and ionic strength have a minor effect on the mass transfer coefficient. A more elaborate calculation that describes the coupled mass transfer of solutes that include electrostatic interactions would be useful to study the mass transport of protein at different values of pH and ionic strength but again would require many more parameters that are not easily assessed independently.

### 4.4. Utilization of the General Rejection Model for Describing Protein Transport through UF

We showed that the rejection of a protein (BSA) is dependent on pH, ionic strength, and TMP. The rejection as a function of fluxes can be described for each pH and ionic strength condition. Membrane users can use the presented approach to describe the rejection of a charge solute and therewith to optimize a concentration process in order to obtain a high flux and high rejection. The concentration process can be optimized further by using a cascade of membrane systems.

The model can be potentially used to describe the kinetics and thermodynamics influence on solute rejection in a novel system. Common approaches to improve the performance of the separation include the development of new membrane materials and modification of the system configuration. Modification of the membrane surface can be performed to reduce the fouling of the membrane [32,33], and spacer design may help in the reduction in concentration polarization [34,35], or by using an extension to the UF system such as sonication, which induces strong local turbulence and enhances the effect of the crossflow [36]. For these systems, the estimation of mass transfer coefficients cannot be performed with Sherwood relations that are based on the conventional crossflow and system geometry. In those cases, the approach that we take can be used, which will help in analyzing the role of the kinetics ( $k_{dbl,i}$ and/or $k_{m,i}$ ) and thermodynamics ( $\sigma_{i}$ ) of solute rejection in the system. Even though the diffusion equation we propose is based on experimental parameterization, it is suitable for easily determining the effects of different process settings, such as transmembrane pressure and the crossflow velocity.

UF is traditionally used to separate a protein from low molecular weight components, such as sugars and salts, or to remove solvents (diafiltration or concentration modes). However, we are mostly interested in the separation of different proteins relative to each other. Separation based on MW is often difficult because many proteins have similar MWs [37]. Our measurements indicate that using a membrane with a relatively high MWCO (in this case 300 kDa) can still lead to appreciable retention, and most importantly, can be adapted by changing the conditions, such as the pH and the ionic strength. This makes the system suitable for separating (or fractionating) proteins. By choosing the conditions such that their charge would be different, one could adapt their respective retentions to be different. For example, by making sure that the pH is far away from the pI of a first protein but further from the pI of a second protein, one would have higher retention of the second protein compared with that of the first protein [4,8]. The ionic strength can then be used to optimize the difference between the two retentions for further design of the overall separation system. This is expected to be useful for designing the fractionation and purification processes of soluble protein mixtures, such as whey proteins (e.g., α-lactalbumin and β-lactoglobulin separation) or egg whites (e.g., lysozyme enrichment).

## 5. Conclusions

A general approach was presented to describe protein rejection through an open UF membrane. A solute flux equation based on solute advection and diffusion was used to derive a general rejection equation and advection-dominated rejection equation. Although the results show that for pH 7, diffusion through the membrane is not significant (*p* value > 0.05), we cannot rule out the role of diffusion at pH 4.9.

The results indicate that the retention of BSA is not caused by direct size exclusion because the MWCO of the membrane used is much larger than the MW of BSA. Instead, electrostatic interactions are probably dominant. This is supported by the finding that the sieving coefficient is dependent on the pH and ionic strength, decreasing from 0.999 to 0.973 with an increase in the ionic strength, and higher at the pI of the protein compared with pH 7. An additional effect that may partly explain the high sieving coefficient of BSA at pH 7 and low ionic strength is oligomerization, which enhances the retention.

The mass transfer coefficient in the polarization layer was more or less independent of the pH and ionic strength in our system. Estimations from Sherwood relations underestimate the value of the mass transfer coefficient and result in inaccurate rejection prediction (adj-*r*^2^ ≤ 0.5). This may be due to the suction effect because we used an open UF membrane, allowing for relatively high transmembrane fluxes.

Overall, the fact that the retention of the membrane is easily adapted by the conditions shows that our system is suitable for protein fractionation. Proteins with similar MW but different electrostatic characteristics (charge number, pI) will experience different retentions.

## Figures and Tables

**Table 1:** Membrane specification.

Membrane Specification	
Membrane name	LX
Model	1812 F
MWCO (declared by manufacturer)	300 kDa
Membrane area	0.334 m^2^
Spacer height	7.8 × 10^−4^ m (31 mil)

MWCO, molecular weight cutoff

**Table 2:** HPLC column specifications and processing conditions.

System	Protein
Column type	TSKGel Extended G2000-G3000SWXL
Column size	300 × 7.8 mm
Temperature	30 °C
Eluent	30% acetonitrile in Milli-Q with 0.1% trifluoroacetic acid
Eluent flow rate	1.5 mL/min
Detector	UV (214 nm)

**Table 3:** Comparison between the general rejection and advection-dominated rejection equations with F test.

pH	Ionic Strength (M)	Adj-*r*^2^ Equation (8)	Adj-*r*^2^ Equation (9)	F Test	*p* Value
7	0	0.38	0.42	0.067	0.80
	0.08	0.99	0.98	5.200	0.15
	0.15	0.99	0.99	5.512	0.14
	0.2	0.95	0.96	−0.000	1.00
4.9	0	0.53	0.55	0.575	0.46
	0.2	0.70	0.58	0.388	0.64

A *p* value <0.05 indicates a significant difference based on the 95% confidence interval.

**Table 5:** Comparison between 2 parameters versus 1 parameter (averaged $k_{dbl}$ from fitting and calculated via Sherwood relations) advection-dominated rejection equation.

pH	Ionic Strength (M)	Mean Fitted		Schock and Miquel [25]		Shi [27]		GL [16]		HH [16]		Bandini and Morelli [26]	
		**F Test**	***p*** **Val**	**F Test**	***p*** **Val**	**F Test**	***p*** **Val**	**F Test**	***p*** **Val**	**F Test**	***p*** **Val**	**F Test**	***p*** **Val**
7	0	0.60	0.45	0.81	0.38	4.86	0.04 *	6.45	0.02 *	8.50	0.01 *	15.46	0.00 *
	0.08	1.17	0.35	36.96	0.01 *	108.00	0.00 *	125.01	0.00 *	140.52	0.00 *	263.66	0.00 *
	0.15	8.06	0.06	158.55	0.00 *	549.50	0.00 *	654.58	0.00 *	47803	0.00 *	995.34	0.00 *
	0.2	1.98	0.19	53.41	0.00 *	182.75	0.00 *	217.32	0.00 *	250.31	0.00 *	272.30	0.00 *
4.9	0	0.25	0.62	1.38	0.26	5.21	0.04 *	6.37	0.03 *	7.67	0.02 *	16.20	0.00 *
	0.2	1.66	0.32	8.40	0.10	15.64	0.06	17.37	0.05	19.01	0.04 *	4.93	0.16

GL, Graetz–Leveque; HH, Harriott–Hamilton. (*) Significant differences between models with 2 and 1 parameters (*p* ≤ 0.05), checking if additional parameter can improve the quality of the model prediction.

**Table 6:** Comparison of $\sigma_{i}$ with Sherwood relations.

		Mean Fitted		Schock and Miquel [25]		Shi [27]		GL [16]		HH [16]		Bandini and Morelli [26]	
**pH**	**I (M)**	$\sigma_{i}$	**Adj** ***r*** **^2^**	** $\sigma_{i}$ **	**Adj** ***r*** **^2^**	** $\sigma_{i}$ **	**Adj** ***r*** **^2^**	** $\sigma_{i}$ **	**Adj** ***r*** **^2^**	** $\sigma_{i}$ **	**Adj** ***r*** **^2^**	** $\sigma_{i}$ **	**Adj** ***r*** **^2^**
7	0	0.9993	0.42	0.9999	0.42	1.0000	0.31	1.0000	0.26	1.0000	0.21	0.9975	0.01
	0.08	0.9935	0.98	0.9988	0.86	0.9999	0.60	1.0000	0.54	1.0000	0.48	0.9758	0.04
	0.15	0.9686	0.98	0.9936	0.84	0.9994	0.47	0.9998	0.37	1.0000	0.27	0.8923	0.04
	0.2	0.9646	0.95	0.9933	0.79	0.9996	0.34	0.9999	0.22	1.0000	−15.30	0.8903	0.03
4.9	0	0.9977	0.57	0.9995	0.54	1.0000	0.42	1.0000	0.36	0.9999	0.32	0.9919	0.02
	0.2	0.9916	0.55	0.9980	−0.42	0.9998	−1.41	0.9999	−1.69	1.0002	−1.87	0.9696	0.05

## Data Availability

The data presented in this study are contained within the article.

## Nomenclatures

Abbreviations:	
BSA	Bovine serum albumin
ED	Electrodialysis
HPLC	High performance liquid chromatography
MWCO	Molecular weight cut off
NF	Nanofiltration
RO	Reverse Osmosis
UF	Ultrafiltration
Symbols:	
*A*	Dimensionless fitted parameter for Sherwood relation [-]
$c_{b}$	Concentration in retentate bulk [mol m^−3^]
$c_{m}$	Concentration at membrane interface at liquid phase [mol m^−3^]
$c_{mem}$	Concentration in the membrane [mol m^−3^]
$c_{p}$	Concentration in permeate [mol m^−3^]
$c_{x=0}$	Concentration at membrane pore entrance [mol m^−3^]
$c_{x=\Delta x}$	Concentration at membrane pore exit [mol m^−3^]
*D*	Diffusion coefficient [m^2^ s^−1^]
$D_{m}$	Diffusion coefficient in the membrane [m^2^ s^−1^]
df	Degree of freedom [-]
$d_{h}$	Hydraulic diameter [m]
*h*	height of channel [m]
$J_{i}$	Solute flux [mol m^−2^ s^−1^]
$K_{c}$	Convective hindrance factor [-]
$K_{d}$	Diffusive hindrance factor [-]
$K_{f}$	Friction factor [-]
$k_{dbl}$	Mass transfer coefficient at concentration polarization layer [m s^−1^]
$k_{m}$	Mass transfer coefficient in the membrane [m s^−1^]
*L*	Length of membrane module [m]
*m*	Dimensionless fitted parameter for Sherwood relation [-]
*n*	Dimensionless fitted parameter for Sherwood relation [-]
$Pe_{d}$	Peclet number at concentration polarization layer [-]
$Pe_{m}$	Peclet number in the membrane [-]
Re	Reynolds number [-]
$R_{obs}$	Observed rejection [-]
$Pe_{d}$	Peclet number at concentration polarization layer [-]
Sc	Schmidt number [-]
Sh	Sherwood number [-]
SS	Sum of squared residuals [-]
$S_{v,p}$	Specific surface of the spacer [m^−1^]
$v_{r}$	Cross flow velocity [m s^−1^]
$v_{w}$	Volumetric flux [m s^−1^]
*x*	coordinate for flow direction [m]
Greek letters:	
$\Delta_{x}$	Difference in location [m]
$\delta_{pol}$	Thickness of polarization layer [m]
$\delta_{mem}$	Thickness of membrane active layer [m]
ε	Spacer porosity [-]
η	Solution viscosity [Pa s]
Φ	Partition coefficient [-]
ρ	Solution density [kg m^−3^]
σ	Sieving coefficient [-]

## Appendix A. Derivation of the Starov Equation

### Appendix A.1. Derivation of the General Solute Rejection Equation

The general rejection equation (Equation (8)) from Starov and Churaev [15] was obtained by combining the solution–friction model for both the concentration polarization layer and the membrane. The solution–friction model was integrated in −Pe form for consistency with Equation (8):

$$-\frac{1}{K_{d,i}D_{m,i}}\int_{0}^{\Delta x}dx=\int_{c_{x=0}}^{c_{x=\Delta x}}\frac{1}{J_{i}-K_{c,i}C_{mem,i}v_{w}}dC_{mem,i}$$

$$-\frac{1}{K_{d,i}D_{m,i}}\int_{0}^{\Delta x}dx=\int_{c_{x=0}}^{c_{x=\Delta x}}\frac{1}{J_{i}-K_{c,i}C_{mem,i}v_{w}}dC_{mem,i}$$

$$-\frac{\left(\Delta \times -0\right)}{K_{d,i}D_{m,i}}=\left(-\frac{1}{K_{c,i}v_{w}}\ln\frac{J_{i}-K_{c,i}c_{x=0}v_{w}}{J_{i}-K_{c,i}c_{x=\Delta x}v_{w}}\right)$$

$$-\frac{K_{c,i}v_{w}\Delta x}{K_{d,i}D_{m,i}}=\ln\frac{J_{i}-K_{c,i}c_{x=0}v_{w}}{J_{i}-K_{c,i}c_{x=\Delta x}v_{w}}$$

By using the Peclet number and the mass transfer coefficient, the solute flux relation through the membrane can also expressed as:

(A1)
$$J_{i}=\frac{K_{c,i}\Phi_{i}v_{w}\left(c_{p,i}\exp\left(-Pe_{m,i}\right)-c_{m,i}\right)}{\exp(-Pe_{m,i})-1}$$

and the expression for the solute flux in the polarization layer is:

(A2)
$$J_{i}=\frac{v_{w}\left(c_{m,i}\exp\left(-Pe_{d,i}\right)-c_{b,i}\right)}{\exp(-Pe_{d,i})-1}$$

The relation for $c_{m,i}$ can be obtained from the expression for the solute flux in the membrane and the overall solute flux relation $J_{i}=v_{w}c_{p,i}$ :

(A3)
$$c_{m,i}=c_{p,i}\exp\left(-Pe_{m,i}\right)-c_{p,i}\frac{\exp(-Pe_{m,i})-1}{K_{c,i}\Phi_{i}}$$

This membrane surface concentration relation was then used to substitute $c_{m,i}$ in Equation (A2):

(A4)
$$J_{i}=v_{w}\frac{\left(c_{p,i}\exp\left(-Pe_{m,i}\right)-c_{p,i}\frac{\exp(-Pe_{m,i})-1}{K_{c,i}\Phi_{i}}\right)\exp\left(-Pe_{d,i}\right)-c_{b,i}}{\exp(-Pe_{d,i})-1}$$

and because $J_{i}=c_{p,i}v_{w}$ , the volumetric flux $v_{w}$ can be canceled out:

$$\begin{array}{c} c_{p,i}\exp\left(-Pe_{d,i}\right)-c_{p_{i}}=\\ \left(c_{p,i}\exp\left(-Pe_{m,i}\right)-c_{p,i}\frac{\exp(-Pe_{m,i})-1}{K_{c,i}\Phi_{i}}\right)\exp\left(-Pe_{d,i}\right)-c_{b,i} \end{array}$$

Divide the equation with permeate concentration $c_{p,i}$ :

$$\begin{array}{c} \exp(-Pe_{d,i})-1=\\ \left(\exp\left(-Pe_{m,i}\right)-\frac{\exp(-Pe_{m,i})-1}{K_{c,i}\Phi_{i}}\right)\exp\left(-Pe_{d,i}\right)-\frac{c_{b,i}}{c_{p,i}} \end{array}$$

Expanding and rearranging the equation results in the ratio of the retentate bulk concentration to the permeate concentration:

(A5)
$$\begin{array}{c} \frac{c_{b,i}}{c_{p,i}}=1+\frac{\exp(-Pe_{d,i})}{K_{c,i}\Phi_{i}}-\frac{\exp\left(-Pe_{m,i}\right)\exp\left(-Pe_{d,i}\right)}{K_{c,i}\Phi_{i}}\\ -\exp\left(-Pe_{d,i}\right)+\exp\left(-Pe_{d,i}\right)\exp\left(-Pe_{m,i}\right) \end{array}$$

Because $R_{obs,i}=1-c_{p,i}/c_{b,i}$ , the previous equation has to be inverted. The equation was modified by multiplying by $K_{c,i}\Phi_{i}/K_{c,i}\Phi_{i}$ :

$$\begin{array}{c} \frac{c_{b,i}}{c_{p,i}}=\frac{K_{c,i}\Phi_{i}}{K_{c,i}\Phi_{i}}+\frac{\exp(-Pe_{d,i})}{K_{c,i}\Phi_{i}}-\frac{\exp\left(-Pe_{m,i}\right)\exp\left(-Pe_{d,i}\right)}{K_{c,i}\Phi_{i}}\\ -\exp\left(-Pe_{d,i}\right)\frac{K_{c,i}\Phi_{i}}{K_{c,i}\Phi_{i}}+\exp\left(-Pe_{d,i}\right)\exp\left(-Pe_{m,i}\right)\frac{K_{c,i}\Phi_{i}}{K_{c,i}\Phi_{i}} \end{array}$$

and by using the $R_{obs,i}$ definition:

$$\begin{array}{c} R_{obs,i}=1-\frac{K_{c,i}\Phi_{i}}{K_{c,i}\Phi_{i}+\exp\left(-Pe_{d,i}\right)-\exp\left(-Pe_{m,i}\right)\exp\left(-Pe_{d,i}\right)-\cdots }\\ \cdots \frac{}{\exp\left(-Pe_{d,i}\right)K_{c,i}\Phi_{i}+\exp\left(-Pe_{d,i}\right)\exp\left(-Pe_{m,i}\right)K_{c,i}\Phi_{i}} \end{array}$$

Collecting $\exp(-Pe_{d,i})$ in the denominator gives:

$$\begin{array}{c} R_{obs,i}=1-\frac{K_{c,i}\Phi_{i}}{K_{c,i}\Phi_{i}+\exp\left(-Pe_{d,i}\right)(1-\exp\left(-Pe_{m,i}\right)-\cdots }\\ \cdots \frac{}{K_{c,i}\Phi_{i}+\exp\left(-Pe_{m,i}\right)K_{c,i}\Phi_{i})} \end{array}$$

The denominator can be simplified further with factorization:

$$R_{obs,i}=1-\frac{K_{c,i}\Phi_{i}}{K_{c,i}\Phi_{i}+\exp\left(-Pe_{d,i}\right)\left(1-\exp\left(-Pe_{m,i}\right)\right)\left(1-K_{c,i}\Phi_{i}\right)}$$

Using the $\sigma_{i}=1-K_{c,i}\Phi_{i}$ , $R_{obs,i}$ becomes Equation (8):

(A6)
$$R_{obs,i}=1-\frac{1-\sigma_{i}}{1-\sigma_{i}+\exp\left(-Pe_{d,i}\right)\left(1-\exp\left(-Pe_{m,i}\right)\right))\sigma_{i}}$$

Alternatively, the concentration ratio in Equation (A5) can also be rearranged into the equation available in the literature. Equation (A5) was simplified with factorization:

$$\frac{c_{b,i}}{c_{p,i}}=1+\left(\frac{\exp(-Pe_{d,i})}{K_{c,i}\Phi_{i}}-\exp\left(-Pe_{d,i}\right)\right)\left(1-\exp(-Pe_{m,i})\right)$$

$$\frac{c_{b,i}}{c_{p,i}}=1+\left(\frac{1}{K_{c,i}\Phi_{i}}-1\right)\exp\left(-Pe_{d,i}\right)\left(1-\exp(-Pe_{m,i})\right)$$

Using the definition of the sieving coefficient, $\sigma_{i}=1-K_{c,i}\Phi_{i}$ , and the observed rejection, $R_{obs,i}=1-c_{p,i}/c_{b,i}$ , the last equation can be simplified and inverted, giving the general rejection equation, which involves mass transfer in the concentration layer and through the membrane (Equation (8)):

(A7)
$$R_{obs,i}=1-{\left[1+\left({\left(1-\sigma_{i}\right)}^{-1}-1\right)\cdot \exp\left(-Pe_{d,i}\right)\left(1-\exp\left(-Pe_{m,i}\right)\right)\right]}^{-1}$$

### Appendix A.2. Derivation of the Solute Rejection Equation without Diffusion through the Membrane

Solute flux (Equation (1)) without the diffusion term can be written as:

(A8)
$$J_{i}=K_{c,i}C_{mem,i}v_{w}=K_{c,i}\Phi_{i}C_{m,i}v_{w}$$

The solute flux relation in the membrane (Equation (A8)) can then be combined with the solute flux in the polarization layer (Equation (A9)) and $J_{i}=v_{w}c_{p,i}$ :

(A9)
$$J_{i}=\frac{v_{w}\left(c_{m,i}\exp\left(-Pe_{d,i}\right)-c_{b,i}\right)}{\exp(-Pe_{d,i})-1}$$

The concentration at the membrane interface ( $c_{m,i}$ ) in the solute flux equation in the polarization layer can be substituted:

$$v_{w}c_{p,i}=K_{c,i}\Phi_{i}c_{m,i}v_{w}$$

$$c_{m,i}=\frac{c_{p,i}}{K_{c,i}\Phi_{i}}$$

giving the following equation:

(A10)
$$J_{i}=\frac{v_{w}\left(\frac{c_{p,i}}{K_{c,i}\Phi_{i}}\exp\left(-Pe_{d,i}\right)-c_{b,i}\right)}{\exp(-Pe_{d,i})-1}$$

After simplification and rearrangement, the equation for the rejection of a solute when advection dominated in the membrane can be derived, and the velocity can be canceled out:

$$c_{p,i}=\frac{\frac{c_{p,i}}{K_{c,i}\Phi_{i}}\exp\left(-Pe_{d,i}\right)-c_{b,i}}{\exp(-Pe_{d,i})-1}$$

Dividing both sides of the equation by $c_{b,i}$ gives:

$$\frac{c_{p,i}}{c_{b,i}}=\frac{\frac{c_{p,i}}{c_{b,i}K_{c,i}\Phi_{i}}\exp\left(-Pe_{d,i}\right)}{\exp(-Pe_{d,i})-1}-\frac{1}{\exp(-Pe_{d,i})-1}$$

Collecting the $c_{p,i}/c_{p,i}$ term and rearranging gives:

$$\left(\exp\left(-Pe_{d,i}\right)-1\right)\frac{c_{p,i}}{c_{b,i}}=\frac{\exp(-Pe_{d,i})}{K_{c,i}\Phi_{i}}\frac{c_{p,i}}{c_{b,i}}-1$$

$$\frac{c_{p,i}}{c_{b,i}}=\frac{1}{\frac{\exp(-Pe_{d,i})}{K_{c,i}\Phi_{i}}-\exp\left(-Pe_{d,i}\right)+1}$$

Multiply the denominator by $K_{c,i}\Phi_{i}/K_{c,i}\Phi_{i}$ :

$$\frac{c_{p,i}}{c_{b,i}}=\frac{1}{\frac{\exp(-Pe_{d,i})}{K_{c,i}\Phi_{i}}-\exp\left(-Pe_{d,i}\right)\frac{K_{c,i}\Phi_{i}}{K_{c,i}\Phi_{i}}+\frac{K_{c,i}\Phi_{i}}{K_{c,i}\Phi_{i}}}$$

$$\frac{c_{p,i}}{c_{b,i}}=\frac{K_{c,i}\Phi_{i}}{\exp\left(-Pe_{d,i}\right)-\exp\left(-Pe_{d,i}\right)K_{c,i}\Phi_{i}+K_{c,i}\Phi_{i}}$$

$$\frac{c_{p,i}}{c_{b,i}}=\frac{K_{c,i}\Phi_{i}}{\exp\left(-Pe_{d,i}\right)\left(1-K_{c,i}\Phi_{i}\right)+K_{c,i}\Phi_{i}}$$

The equation was then modified with the definition of rejection and the sieving coefficient:

$$R_{obs,i}=1-\frac{1-\sigma_{i}}{\exp\left(-Pe_{d,i}\right)\left(1-\left(1-\sigma_{i}\right)\right)+\left(1-\sigma_{i}\right)}$$

The resulting equation is the same as Equation (9), derived directly from Equation (8):

(A11)
$$R_{obs,i}=1-\frac{1-\sigma_{i}}{1-\sigma_{i}+\exp\left(-Pe_{d,i}\right)\sigma_{i}}$$

## Appendix B. Data Table

### Appendix B.1. BSA Solution Volumetric Flux and Concentration Data at Different Condition

**Table A1:** Volumetric flux and BSA concentration at different pH and ionic strength.

pH	Ionic	TMP	Flux	SD Flux	$C_{r,\mathrm{BSA}}$	SD	$C_{p,\mathrm{BSA}}$	SD
	**Str (M)**	**(bar)**	**(m^3^ m^−2^ s^−1^)**		**(mg mL^−1^)**	** $C_{r,\mathrm{BSA}}$ **	**(mg mL^−1^)**	** $C_{p,\mathrm{BSA}}$ **
4.9	0	0.2	3.30 × 10^−6^	8.17 × 10^−8^	3.26	4.20 × 10^−2^	8.55 × 10^−3^	3.54 × 10^−4^
4.9	0	0.2	2.99 × 10^−6^	3.72 × 10^−8^	2.98	1.10 × 10^−2^	1.60 × 10^−2^	7.07 × 10^−5^
4.9	0	0.2	2.48 × 10^−6^	3.21 × 10^−8^	3.58	1.97 × 10^−2^	7.05 × 10^−3^	7.07 × 10^−5^
4.9	0	0.5	5.76 × 10^−6^	6.86 × 10^−8^	3.29	1.04 × 10^−2^	9.10 × 10^−3^	4.24 × 10^−4^
4.9	0	0.5	5.30 × 10^−6^	5.97 × 10^−8^	3.04	1.13 × 10^−3^	2.12 × 10^−2^	2.12 × 10^−4^
4.9	0	0.5	4.64 × 10^−6^	1.06 × 10^−7^	3.55	4.17 × 10^−3^	1.95 × 10^−2^	1.41 × 10^−4^
4.9	0	0.8	7.78 × 10^−6^	4.42 × 10^−8^	3.30	6.36 × 10^−4^	1.09 × 10^−2^	1.41 × 10^−4^
4.9	0	0.8	7.23 × 10^−6^	6.49 × 10^−8^	3.02	1.20 × 10^−3^	2.60 × 10^−2^	2.12 × 10^−4^
4.9	0	1.5	1.15 × 10^−5^	4.66 × 10^−8^	3.36	9.40 × 10^−3^	2.70 × 10^−2^	4.24 × 10^−4^
4.9	0	1.5	1.09 × 10^−5^	3.99 × 10^−8^	2.99	6.93 × 10^−3^	4.75 × 10^−2^	8.49 × 10^−4^
4.9	0	1.5	9.60 × 10^−6^	6.30 × 10^−8^	3.70	1.07 × 10^−2^	6.95 × 10^−3^	2.12 × 10^−4^
4.9	0	3	1.71 × 10^−5^	4.31 × 10^−8^	3.41	1.34 × 10^−3^	7.84 × 10^−2^	5.66 × 10^−4^
4.9	0	3	1.62 × 10^−5^	3.80 × 10^−8^	3.13	9.90 × 10^−4^	8.86 × 10^−2^	0.00
4.9	0	3	1.45 × 10^−5^	3.30 × 10^−8^	3.67	9.83 × 10^−3^	6.35 × 10^−3^	7.07 × 10^−5^
4.9	0.2	0.2	4.62 × 10^−6^	2.72 × 10^−6^	2.92	1.15 × 10^−2^	9.04 × 10^−2^	4.95 × 10^−4^
4.9	0.2	0.5	5.04 × 10^−6^	4.76 × 10^−8^	3.78	1.83 × 10^−2^	6.02 × 10^−2^	2.83 × 10^−4^
4.9	0.2	1.5	9.95 × 10^−6^	5.82 × 10^−8^	3.77	1.30 × 10^−2^	1.04 × 10^−1^	7.07 × 10^−4^
4.9	0.2	3	1.46 × 10^−5^	7.83 × 10^−8^	3.84	2.26 × 10^−2^	1.91 × 10^−1^	1.41 × 10^−4^
7	0	0.2	3.37 × 10^−6^	2.98 × 10^−8^	2.92	8.20 × 10^−3^	3.55 × 10^−3^	7.07 × 10^−5^
7	0	0.2	2.77 × 10^−6^	5.35 × 10^−8^	3.57	3.24 × 10^−2^	9.15 × 10^−3^	7.07 × 10^−5^
7	0	0.2	2.30 × 10^−6^	4.83 × 10^−8^	3.65	5.49 × 10^−2^	2.85 × 10^−3^	3.54 × 10^−4^
7	0	0.2	3.27 × 10^−6^	4.34 × 10^−8^	3.43	5.22 × 10^−2^	5.50 × 10^−3^	0.00
7	0	0.2	3.50 × 10^−6^	5.27 × 10^−8^	3.53	5.40 × 10^−2^	0.00	0.00
7	0	0.2	2.85 × 10^−6^	3.86 × 10^−8^	3.54	4.98 × 10^−2^	0.00	0.00
7	0	0.5	6.00 × 10^−6^	4.47 × 10^−8^	3.00	1.57 × 10^−2^	3.70 × 10^−3^	1.41 × 10^−4^
7	0	0.5	5.47 × 10^−6^	7.81 × 10^−8^	3.75	4.27 × 10^−2^	5.40 × 10^−3^	1.41 × 10^−4^
7	0	0.5	6.02 × 10^−6^	6.15 × 10^−8^	3.44	4.24 × 10^−4^	9.50 × 10^−4^	7.07 × 10^−5^
7	0	0.5	6.20 × 10^−6^	6.88 × 10^−8^	3.52	6.07 × 10^−2^	0.00	0.00
7	0	0.8	8.07 × 10^−6^	4.47 × 10^−8^	2.99	1.29 × 10^−2^	5.90 × 10^−3^	1.41 × 10^−4^
7	0	0.8	7.41 × 10^−6^	5.57 × 10^−8^	3.86	6.91 × 10^−2^	5.95 × 10^−3^	7.07 × 10^−5^
7	0	0.8	8.30 × 10^−6^	8.09 × 10^−8^	3.50	8.63 × 10^−3^	0.00	0.00
7	0	0.8	8.17 × 10^−6^	7.27 × 10^−8^	3.45	4.74 × 10^−2^	2.00 × 10^−3^	1.41 × 10^−4^
7	0	1.5	1.17 × 10^−5^	5.70 × 10^−8^	3.15	3.04 × 10^−3^	1.15 × 10^−2^	7.07 × 10^−5^
7	0	1.5	1.13 × 10^−5^	8.18 × 10^−8^	3.95	5.60 × 10^−2^	9.75 × 10^−3^	2.12 × 10^−4^
7	0	1.5	1.24 × 10^−5^	6.78 × 10^−8^	3.54	8.20 × 10^−3^	1.55 × 10^−3^	7.07 × 10^−5^
7	0	1.5	1.16 × 10^−5^	7.99 × 10^−8^	3.50	5.42 × 10^−2^	7.20 × 10^−3^	2.83 × 10^−4^
7	0	3	1.72 × 10^−5^	6.82 × 10^−8^	3.18	6.22 × 10^−3^	5.16 × 10^−2^	0.00
7	0	3	1.69 × 10^−5^	8.17 × 10^−8^	4.08	4.57 × 10^−2^	2.97 × 10^−2^	2.12 × 10^−4^
7	0	3	1.81 × 10^−5^	9.90 × 10^−8^	3.59	3.18 × 10^−3^	1.75 × 10^−3^	7.07 × 10^−5^
7	0	3	1.66 × 10^−5^	1.28 × 10^−7^	3.44	4.96 × 10^−2^	3.01 × 10^−2^	7.07 × 10^−5^
7	0.08	0.2	3.32 × 10^−6^	8.93 × 10^−8^	2.99	2.09 × 10^−2^	3.54 × 10^−2^	2.83 × 10^−4^
7	0.08	0.5	5.85 × 10^−6^	3.95 × 10^−8^	3.11	2.43 × 10^−2^	3.54 × 10^−2^	1.41 × 10^−4^
7	0.08	0.8	7.84 × 10^−6^	3.05 × 10^−8^	3.17	2.06 × 10^−2^	4.23 × 10^−2^	2.12 × 10^−4^
7	0.08	1.5	1.14 × 10^−5^	5.22 × 10^−8^	3.25	5.44 × 10^−3^	8.33 × 10^−2^	2.83 × 10^−4^
7	0.08	3	1.74 × 10^−5^	8.27 × 10^−8^	3.20	2.70 × 10^−2^	1.95 × 10^−1^	1.06 × 10^−3^
7	0.15	0.2	3.63 × 10^−6^	5.54 × 10^−8^	3.61	6.08 × 10^−3^	1.71 × 10^−1^	4.95 × 10^−4^
7	0.15	0.5	6.06 × 10^−6^	5.43 × 10^−8^	3.61	9.12 × 10^−3^	2.00 × 10^−1^	1.27 × 10^−3^
7	0.15	0.8	8.10 × 10^−6^	3.17 × 10^−8^	3.69	2.55 × 10^−2^	2.60 × 10^−1^	1.06 × 10^−3^
7	0.15	1.5	1.18 × 10^−5^	6.07 × 10^−8^	3.71	2.57 × 10^−2^	4.63 × 10^−1^	3.75 × 10^−3^
7	0.15	3	1.74 × 10^−5^	7.23 × 10^−8^	3.67	1.82 × 10^−2^	9.20 × 10^−1^	2.62 × 10^−3^
7	0.2	0.2	3.41 × 10^−6^	6.15 × 10^−8^	3.59	4.17 × 10^−3^	2.47 × 10^−1^	1.98 × 10^−3^
7	0.2	0.2	3.22 × 10^−6^	4.76 × 10^−8^	3.43	1.47 × 10^−2^	1.22 × 10^−1^	2.33 × 10^−3^
7	0.2	0.2	2.80 × 10^−6^	1.87 × 10^−8^	3.56	3.93 × 10^−2^	4.32 × 10^−2^	8.49 × 10^−4^
7	0.2	0.5	6.08 × 10^−6^	6.35 × 10^−8^	3.59	2.27 × 10^−2^	3.04 × 10^−1^	7.07 × 10^−5^
7	0.2	0.5	5.41 × 10^−6^	6.39 × 10^−8^	3.61	4.26 × 10^−2^	1.39 × 10^−1^	1.77 × 10^−3^
7	0.2	0.8	8.12 × 10^−6^	7.46 × 10^−8^	3.71	2.60 × 10^−2^	3.76 × 10^−1^	5.66 × 10^−4^
7	0.2	0.8	7.15 × 10^−6^	5.31 × 10^−8^	3.61	3.33 × 10^−2^	2.01 × 10^−1^	1.91 × 10^−3^
7	0.2	1.5	1.19 × 10^−5^	6.28 × 10^−8^	3.75	1.28 × 10^−2^	5.41 × 10^−1^	1.27 × 10^−3^
7	0.2	1.5	1.03 × 10^−5^	1.07 × 10^−7^	3.61	3.68 × 10^−2^	4.36 × 10^−1^	2.76 × 10^−3^
7	0.2	3	2.00 × 10^−5^	1.79 × 10^−7^	3.58	1.19 × 10^−2^	1.26	2.12 × 10^−4^
7	0.2	3	1.51 × 10^−5^	1.49 × 10^−7^	3.57	4.16 × 10^−2^	7.42 × 10^−1^	5.44 × 10^−3^

### Appendix B.2. Fitted Parameters of the General Rejection and Advection-Dominated Rejection Equations

**Table A2:** Fitted parameters of the general rejection equation (Equation (8)). Different lower-case letters indicate a significant difference for each parameter.

pH	I (M)	$\sigma_{i}$	95% CI	$k_{\mathrm{dbl},i}$	95% CI	$k_{m,i}$	95% CI	Adj-*r^2^*	df
7	0	0.999 ^a^	1.14 × 10^−4^	5.55 × 10^−6 a^	6.21 × 10^−6^	2.32 × 10^−6 a^	2.24 × 10^−5^	0.387	19
	0.08	0.997 ^a^	7.15 × 10^−3^	5.91 × 10^−6 a^	3.88 × 10^−6^	4.30 × 10^−6 a^	1.41 × 10^−5^	0.994	2
	0.15	0.979 ^b^	9.08 × 10^−3^	6.32 × 10^−6 a^	1.08 × 10^−6^	2.34 × 10^−6 a^	3.07 × 10^−6^	0.998	2
	0.2	0.973 ^b^	9.00 × 10^−3^	6.70 × 10^−6 a^	9.79 × 10^−7^	5.38 × 10^−8 a^	0	0.966	9
4.9	0	0.999 ^ab^	0.36	4.00 × 10^−6 a^	5.28 × 10^−4^	6.97 × 10^−5 a^	0.33	0.532	11
	0.2	0.999 ^a^	6.39 × 10^−5^	5.28 × 10^−6 a^	3.39 × 10^−6^	1.29 × 10^−4 a^	1.70 × 10^−4^	0.704	2

CI, confidence interval; df, degrees of freedom.

**Table A3:** Fitted parameters of the advection-dominated rejection equation (Equation (9)). Different lower-case letters indicate a significant difference for each parameter.

pH	I (M)	$\sigma_{i}$	95% CI	$k_{\mathrm{dbl},i}$	95% CI	Adj-*r*^2^	df
7	0	0.999 ^a^	7.32 × 10^−4^	5.71 × 10^−6^ ^a^	4.03 × 10^−6^	0.415	20
	0.08	0.995 ^b^	2.60 × 10^−3^	6.78 × 10^−6 a^	1.55 × 10^−6^	0.986	3
	0.15	0.977 ^c^	5.90 × 10^−3^	6.62 × 10^−6 a^	7.45 × 10^−7^	0.997	3
	0.2	0.973 ^c^	9.00 × 10^−3^	6.69 × 10^−6 a^	9.78 × 10^−7^	0.962	9
4.9	0	0.998 ^ab^	2.30 × 10^−3^	6.27 × 10^−6 a^	3.85 × 10^−6^	0.549	12
	0.2	0.985 ^abc^	2.64 × 10^−2^	1.19 × 10^−5 a^	2.22 × 10^−5^	0.591	2

CI, confidence interval; df, degrees of freedom.

**Table A4:** Fitted parameters of advection-dominated rejection equation (Equation (9)) with averaged mass transfer coefficient (7.34 × 10^−6^ m/s). Different lower-case letters indicate a significant difference.

pH	I (M)	$\sigma_{i}$	95% CI	Adj-*r*^2^	df
7	0	0.999 ^a^	2.70 × 10^−4^	0.420	21
	0.08	0.994 ^b^	8.43 × 10^−4^	0.978	4
	0.15	0.969 ^c^	4.10 × 10^−3^	0.981	4
	0.2	0.965 ^c^	4.40 × 10^−3^	0.954	10
4.9	0	0.998 ^a^	7.33 × 10^−4^	0.570	13
	0.2	0.992 ^b^	4.00 × 10^−3^	0.547	3

CI, confidence interval; df, degrees of freedom.
